# Extracellular Vesicles Secreted by Glioma Stem Cells Are Involved in Radiation Resistance and Glioma Progression

**DOI:** 10.3390/ijms23052770

**Published:** 2022-03-02

**Authors:** Chenkai Ma, Hong P. T. Nguyen, Jordan J. Jones, Stanley S. Stylli, Clarissa A. Whitehead, Lucy Paradiso, Rodney B. Luwor, Zammam Areeb, Eric Hanssen, Ellie Cho, Ulrich Putz, Andrew H. Kaye, Andrew P. Morokoff

**Affiliations:** 1 Department of Surgery, The University of Melbourne, Melbourne, VIC 3052, Australia; chenkaim@student.unimelb.edu.au (C.M.); hong.nguyen1@unimelb.edu.au (H.P.T.N.); jones.jordanj@gmail.com (J.J.J.); sstylli@unimelb.edu.au (S.S.S.); cwhitehead@student.unimelb.edu.au (C.A.W.); lucialp@unimelb.edu.au (L.P.); rluwor@unimelb.edu.au (R.B.L.); z.areeb@student.unimelb.edu.au (Z.A.); andrewk@hadassah.org.il (A.H.K.); 2 Department of Neurosurgery, The Royal Melbourne Hospital, Melbourne, VIC 3052, Australia; 3 Bio21 Ian Holmes Imaging Center, Department of Biochemistry and Pharmacology, Bio21 Institute, The University of Melbourne, Melbourne, VIC 3052, Australia; ehanssen@unimelb.edu.au; 4 Biological Optical Microscopy Platform, The University of Melbourne, Grattan Street, Melbourne, VIC 3010, Australia; ellie.cho@unimelb.edu.au; 5 The Florey Institute of Neuroscience, Royal Parade, Melbourne, VIC 3052, Australia; ulrich.putz@florey.edu.au; 6 Department of Neurosurgery, Hadassah Hebrew University Hospital, Jerusalem 91120, Israel

**Keywords:** glioblastomas, exosomes, EVs, stem cells, glioma stem cells, miRNAs, PTEN, profiling

## Abstract

Glioblastoma is the most aggressive brain tumour with short survival, partly due to resistance to conventional therapy. Glioma stem cells (GSC) are likely to be involved in treatment resistance, by releasing extracellular vesicles (EVs) containing specific molecular cargoes. Here, we studied the EVs secreted by glioma stem cells (GSC-EVs) and their effects on radiation resistance and glioma progression. EVs were isolated from 3 GSCs by serial centrifugation. NanoSight measurement, cryo-electron microscopy and live imaging were used to study the EVs size, morphology and uptake, respectively. The non-GSC glioma cell lines LN229 and U118 were utilised as a recipient cell model. Wound healing assays were performed to detect cell migration. Colony formation, cell viability and invadopodium assays were conducted to detect cell survival of irradiated recipient cells and cell invasion post GSC-EV treatment. NanoString miRNA global profiling was used to select for the GSC-EVs’ specific miRNAs. All three GSC cell lines secreted different amounts of EVs, and all expressed consistent levels of CD9 but different level of Alix, TSG101 and CD81. EVs were taken up by both LN229 and U118 recipient cells. In the presence of GSC-EVs, these recipient cells survived radiation exposure and initiated colony formation. After GSC-EVs exposure, LN229 and U118 cells exhibited an invasive phenotype, as indicated by an increase in cell migration. We also identified 25 highly expressed miRNAs in the GSC-EVs examined, and 8 of these miRNAs can target PTEN. It is likely that GSC-EVs and their specific miRNAs induced the phenotypic changes in the recipient cells due to the activation of the PTEN/Akt pathway. This study demonstrated that GSC-EVs have the potential to induce radiation resistance and modulate the tumour microenvironment to promote glioma progression. Future therapeutic studies should be designed to interfere with these GSC-EVs and their specific miRNAs.

## 1. Introduction

Glioblastoma is the most common and aggressive type of brain cancer with poor median survival of around one year. Despite maximal safe surgical resection and a combination of radiotherapy and chemotherapy [1,2], tumour recurrence is almost always seen, probably due to tumour heterogeneity both at a phenotypic and a molecular level [3]. The prognosis remains poor, despite a gross total resection at the first and at the second surgery (recurrence); however, overall survival is better in patients who had second surgery with local recurrence compared to non-local recurrence [4], as well as in those with a combination of bevacizumab and fractionated stereotactic radiotherapy (FSRT) treatment [5]. 

A small subpopulation of stem-like cancer cells (glioma stem cells, GSCs) is thought to be associated with resistance and subsequent repopulation of glioma cells post treatment [6,7,8]. The plasticity of differentiation or trans-differentiation of GSCs is regarded as one of the key drivers of recurrence [9,10]. However, the mechanisms of how GSCs facilitate their non-GSC glioma cells to progress are not fully elucidated [11].

Intercellular communication is emerging as an important feature of cancer and its surrounding microenvironment [12]. This communication is likely to be mediated by nano-size extracellular vesicles (EVs) and smaller vesicles of 30–100 nm called exosomes [13,14]. Several studies have demonstrated that exosomes contain specific DNA, RNA, miRNA and proteins that can cause changes in gene expression in recipient cells [15,16,17]. EV-mediated intra-tumour communication from stem cells to non-stem cells may also serve to promote a more aggressive cancer phenotype [18]. Glioma-derived EVs are also known to play a critical role in immune responses [19,20], stimulation of angiogenesis in surrounding cells under hypoxic conditions [21] and transfer of a resistance phenotype [22,23,24,25,26,27]. In addition, a recent co-culture study of both proneural and mesenchymal phenotypes has demonstrated that EVs were able to modulate and regulate intercellular communication within the spheroid population in vitro. 

Here, we isolated EVs directly from patient-derived glioma stem cells and studied their miRNA cargo and functional effects on the non-GSC glioma cells (LN229 and U118). We showed that GSC-EVs can increase migration, as well as transfer radio-resistance related cargo to recipient cells, possibly via secreting various miRNAs. However, the exact mechanism of how GSC-EVs regulate the glioma cell population and the microenvironment needs to be fully elucidated. 

## 2. Results and Discussion

### 2.1. Exosomes Characterisation and Internalisation

Glioma stem cells (GSCs) derived from three patients (Supplementary Figure S1A), grown as gliomaspheres, were firstly confirmed with the presence of neuronal and stem cell markers (Nestin, GFAP, Oct3/4 and Sox2) prior to isolation of EVs. All three GSC cell lines (MU004, MU020, MU039) expressed stem cells markers, but non-GSC cell lines (LN229 and U118) did not (Supplementary Figure S1B). 

**Figure 1:**
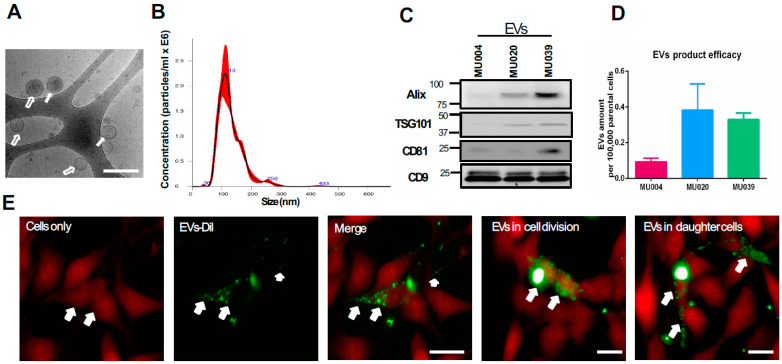
Characteristics of GSC-EVs. (**A**) Cryo-electron microscopy showing round, cup-shaped EVs (GSCs-EVs, white arrows) post differential centrifugation. Scale bar: 200 nm. (**B**) NanoSight tracking analysis of concentration and size distribution of GSC-EVs. (**C**) Western blot analysis of exosomal markers (Alix, TSG101, CD81, CD9) in GSC-EVs (MU004, MU020, MU039). (**D**) Amount of EV production by GSC-EVs. (**E**) Representative images of GSC-EVs uptake and EVs presence in cell division and daughter cells. Red: recipient cell only; green: DiI-labelled EVs. Scale bar 200 um.

Exosomes/EVs (30–150 nm, mode size 106 ± 4.7 nm) with round and membranous cup-shaped morphology were secreted by glioma stem cells, as characterised byusing a cryo-transmission electron microscope and NanoSight distribution (Figure 1A,B). GSC-EV concentrations ranged from 2.1 × 10^8^ particles/mL to 4.8 × 10^8^ particles/mL and were positive for the exosomal markers Alix, TSG101, CD9 and CD81 (Figure 1C). The CD9 expression level was seen to be consistent in EVs of all three GSC lines, while the expression level varied in other markers (Alix, TSG101 and CD81). This is in agreement with other studies [28,29]. All three GSC cell lines produced different amounts of EVs (Figure 1D), possibly reflecting the heterogeneity of the parental tumour cells. Since MU004, MU020 and MU039 belong to proneural, mesenchymal and a mixture of proneural and mesenchymal subtypes, respectively, [11,30], it is likely that the EVs content recapitulates their molecular subtype. MU020 has mesenchymal features [11], indicating that mesenchymal-like GSCs are likely to secret more EVs to modulate the surrounding bulk tumour cells. After characterisation of the GSC-EVs, we then tested whether these GSC-EVs could be taken up by the recipient cells. GSC-EVs could be taken up by recipient cells (LN229 or U118) within 16 h and were transferred to the daughter cells during cell division (Figure 1E and Supplementary Video S1). We also investigated the GSC-EVs storage conditions (fresh or −20 °C storage) and found that they retained their membrane structure and morphology, and that storage did not affect their functions to induce colony formation and cellular migration in LN229 recipient cells (Figure S2A,B).

### 2.2. GSC-EVs Enhance Radiation Resistance in Glioma Cells

**Figure 2:**
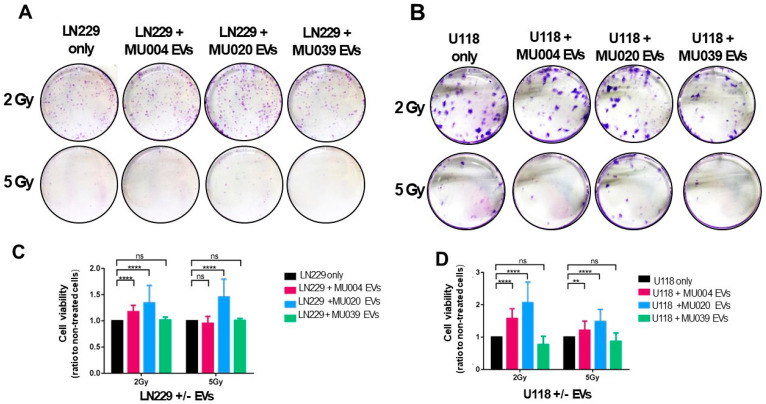
GSCs-EVs transfer radiation resistance. Representative images of colony formation of recipient cells (**A**) LN229 and (**B**) U118 with or without GSC-EVs after exposure to 2 Gy and 5 Gy radiation dosage. Cell viability of (**C**) LN229 and (**D**) U118 cells with and without GSC-EVs post 2 Gy and 5 Gy radiation. Significance: ns—not significant; ** *p* ≤ 0.01; **** *p* ≤ 0.0001.

To test whether GSC-EVs could enhance the radiation resistance of recipient cells, we performed irradiation of recipient cells 6 h prior to GSC-EVs addition, followed by a colony formation assay. All the EVs derived from the three GSC lines could enhance the radiation resistance of LN229 and U118 recipient cells by increasing the colonies formed (Figure 2A,B). More visible colonies were observed with 2 Gy irradiation compared to 5 Gy. Notably, recipient cells still survived irradiation with 5 Gy exposures and formed colonies in the presence of MU020 EVs. Cell viability experiments also confirmed that the presence of EVs resulted in both LN229 and U118 surviving the radiation exposure (Figure 2C,D).

### 2.3. GSC-Derived Exosomes Enhanced Tumour Cell Migration and Spreading 

**Figure 3:**
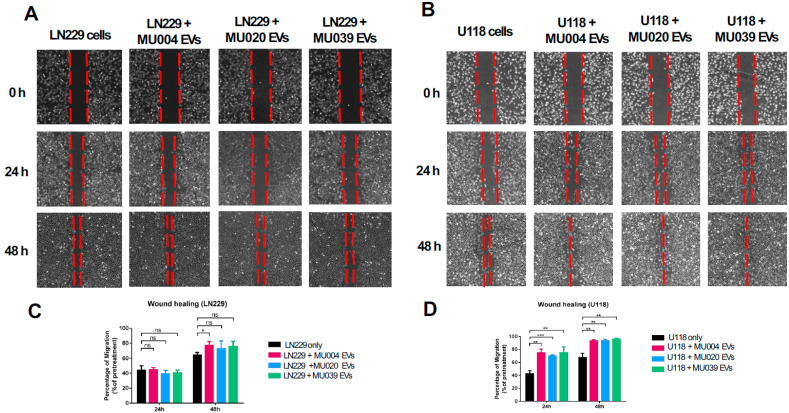
GSC-EVs promote cell migration. Representative phase contrast images of cell migration of (**A**) LN229 cells and (**B**) U118 cells in response to EVs from MU004, MU20 and MU39 GSCs at 24 h and 48 h. Bar graphs show the percentage of migration of (**C**) LN229 cells and (**D**) U118 cells in the presence of GSC-EVs compared to untreated cells. Significance: ns—not significant; * *p* < 0.05; ** *p* ≤ 0.01; *** *p* ≤ 0.001.

Both LN229 and U118 cells migrated faster in the presence of GSC-EVs (Figure 3A,B), with U118 cells migrating fastest (Figure 3B). A significant rate of migration was observed at 48 h but not at 24 h post GSC-EV treatment, in both LN229 and U118 cells, in response to MU004 EVs compared to MU020 EVs and MU039 EVs (Figure 3C,D). 

### 2.4. GSC-EVs Are Involved in Invadopodia and Proliferative Activity

**Figure 4:**
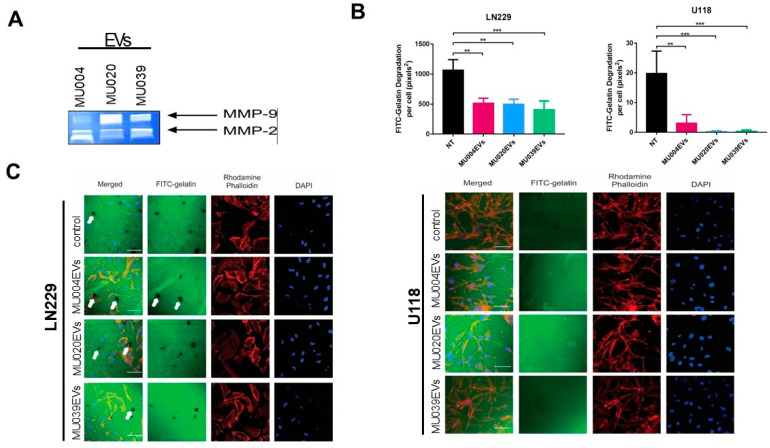
GSC-EVs and invadopodia activity. (**A**) Representative images of GSC-EVs containing invadopodia proteins MMP9 and MMP2. (**B**) Quantification of FITC–gelatin-coated degradation of LN229 and U118 cells after GSC-EVs exposure. Significance: ** *p* ≤ 0.05; *** *p* ≤ 0.01. (**C**) Representative confocal images of FITC–gelatin-coated degradation. White arrows: FITC–gelatin; red: rhodamine/phalloidin; blue: DAPI.

Glioma can be characterised by tumour cell invasion facilitated by invadopodia and associated proteases secretion [31], therefore we then examined whether GSC-EVs contained proteases. Using zymography, MMP-2 and MMP-9 bands were detected in all GSC-EVs (Figure 4A), suggesting the potential role of EVs in extracellular matrix degradation and invasion. MMP-2 expression level was higher in MU004 EVs and MU039 EVs, while MMP-9 level was more prominent in MU020 EVs (Figure 4A). Pre-incubation of the LN229 and U118 cells with GSC-EVs (MU004 EVs, MU020 EVs or MU039 EVs) resulted in a significant reduction in the level of invadopodia-mediated FITC–gelatin degradation compared to the untreated cells (Figure 4B,C). These data confirm our previous findings that the actin-rich invadopodia structures can facilitate matrix degradation and promote glioma cell invasion [32,33] through EV regulation [34]. 

### 2.5. miRNAs Cargoes in EVs Reflect GBM Phenotype

To ascertain whether GSC-EVs contain any miRNAs that may reflect the GBM phenotype, we profiled the GSC-EVs using a NanoString miRNA assay, analysed using nSolver analysis software (v3 NanoString) and the R statistical program package. A list of 25 miRNAs were found in common within EVs derived from three GSC cell lines (Table 1). Of these, 8 miRNAs (mir320e, mir520f-3p, mir363-3p, mir144-4p, mir16-5p, mir495-3p, mir23a-3p, mir155-5p) could target the PTEN pathway when analysed and compared using TargetScan.

Further analysis was performed, and the results compared with the online database “Glioblastoma Bio Discovery Portal, GBM-BioDP”. It was found that 3 miRNAs (hsa-miR-142-3p, hsa-miR-551a and hsa-miR-662) out of the 25 miRNAs were associated with different subtypes of glioblastoma (Supplementary Figure S3A). Hierarchical clustering showed a high correlation of hsa-miR-142-3p with mesenchymal phenotypes of GBM [35] (Supplementary Figure S3B), while hsa-miR551a and hsa-miR612 are seen in both mesenchymal subtypes and classical subtypes. High expression of these 3 miRNAs were also associated with worse survival in the proneural subtype compared to other subtypes of GBM.

In this study, we demonstrated that EVs released by glioma stem cells were taken up by the recipient glioma cells and induced a phenotypic change of the glioma cells, leading them to become more migratory, invasive and radiation-resistant. These changes possibly occur via the transfer of miRNAs contents packaged within the EVs, and thus more research is underway in our laboratory to further investigate the role of these miRNAs in glioma tissue and the signalling pathways involved.

## 3. Discussion

Glioblastoma is the most aggressive form of brain cancer with little or no targeted treatment. It is known that the glioma stem cells are likely to be responsible for treatment resistance and tumour recurrence. Here, in this study, we found that GSCs secreted various numbers of extracellular vesicles (EVs) to modulate the microenvironment and change the tumour cell behaviour. 

We studied three different GSC cell lines (MU004, MU020, MU039) derived directly from patient tissue biopsies and found that various amounts of EVs were secreted and that they expressed different exosomal markers, consistent with the published literature [36]. We found that CD9 protein was visible uniformly across all three GSC lines, which will enable us to use CD9 antibody to uniformly capture and isolate all exosomes from glioma stem cells in future studies. Our results also expand the previous conclusion that CD9 is conservatively expressed in EVs into cancer stem cells, which highlights the utility of CD9 as an EVs marker across variable cell types. 

EVs secreted by tumour cells have demonstrated their ability to remodel the phenotypes of surrounding normal cells in the microenvironment, such as microglia in the brain, into a tumour-accommodating niche [19]. It is also possible that changes may occur in the genetic profiles of primary and recurrence tumours [37] and would be reflected in the EVs cargo contents, which should be further investigated. Our results highlighted that glioma cells can be manipulated by GSC-EVs to enhance progression. EVs were taken up by both LN229 and U118 recipient cells in this study and participated in cell division, suggesting that EVs are heavily involved in cell proliferation and maintenance of tumour cells within the microenvironment. 

In addition, our data demonstrated that in the presence of EVs, the recipient cells can survive radiation exposure, which agrees with studies in the haematopoietic system [38,39]. The fact that both LN229 and U118 recipient cells treated with GSC-EVs were able to form more colonies after radiation indicates that GSCs are not only resistant to therapeutic treatment but can influence their neighbouring cells to coordinate the tumour evolution by releasing EVs [40]. 

It has been established that EVs mediate the transfer of histones, oncogenic species (EGFRvIII) [41], non-coding RNA (miRNA) [20] and tumour suppressors (PTEN) in glioma cells [16] and that therapeutically resistant cancer cells can transfer this resistance to sensitive cancer cells via EVs in renal cancer, breast cancer and colorectal cancer [22,24,42]. Therefore, inhibition of EV secretion or neutralising EV productions in glioma will be investigated further as a potential therapeutic target in our future studies.

We demonstrated that GSC-EVs have the ability to promote glioma cell migration, facilitate matrix degradation and promote invadopodia activity, mostly observed when GSC-EVs were added to LN229 cells. Additionally, the fact that recipient cells in this study survived radiation and formed colonies suggests that EVs play a role in cell proliferation and contribute to the “go or grow” theory of both invasion and proliferation [43,44]. It is possible that the mechanism of the interactions between EVs and the recipient cells can result in a final outcome where the invasive phenotype will have a low proliferative capacity, while the less invasive glioma cells are likely to be associated with high proliferative potential [45,46]. This inverse relationship between invasion and proliferation of glioma cells highlights the dynamic balance of cell survival when confronted with therapeutic stress [47]. It is likely that GSCs can modulate the tumour microenvironment and nearby non-GSC cells into a more proliferative or migratory phenotype by releasing EVs with specific cargo contents. This is evident in studies where GSC-EVs can modulate the activity of mesenchymal stem cells and their miRNA profiles, by mechanisms such as decreasing anti-tumour miRNAs [48] or transferring EGFRvIII molecules to cause a cellular transformation of recipient cells [41], or where EVs release the invasiveness-related proteins into the microenvironment to degrade the extracellular matrix [49]. Our data are also in agreement with a recent study by Pan et al. [44], demonstrating that glioblastoma-derived EVs can promote proliferation, migration and intercellular communication in surrounding tumour cells by manipulating the PI3K-Akt-mTOR pathway. We are setting up an experiment to further investigate the effects of GSC-EVs treatment on pathways involving PTEN, PI3K and Akt. 

It is known that the cargoes in EVs, mainly miRNAs and proteins, can be selectively packaged and transported to recipient cells to modulate their functions [36]. Our NanoString data showed that there were at least 25 highly expressed miRNAs in all three GSC lines and that 8 of the 25 miRNAs (mir320e, mir520f-3p, mir363-3p, mir144-4p, mir16-5p, mir495-3p, mir23a-3p, mir155-5p) have a seed sequence to target PTEN, whilst 3 of the 25 miRNAs (mir612, mir142-3p, mir551a) were found to be common in the Glioblastoma Bio Discovery Portal of the TCGA database. This is in line with previous publications showing that miRNAs packaged within EVs can modulate the intercellular communications within the brain microenvironment [15,50,51,52], as well as playing a role in how the tumour responds to therapy [53]. There were many abundantly expressed miRNAs found in the EVs of T98G cells subjected to invasion [51] and serum EVs [52] involved in glioma progression [54], yet only a few miRNAs were found in common with our study, expressing at low levels (Supplementary Table S2). This is probably due to the type and heterogeneity of the cell lines studied; nevertheless, it indicates that EVs do specifically package certain miRNAs to modulate the tumour microenvironment and the intercellular communication between tumour cells and the surroundings. 

Considering the potential diagnostic ability of circulating miRNAs in glioma and the relatively high proportion of miRNAs in EVs’ RNA cargo [52,55,56,57], we are currently examining these specific miRNAs in glioma tissue and liquid biopsies to identify biomarkers for glioma progression.

We acknowledge that this study only used GSC-EVs from three patients, and we are planning to obtain more EVs from a different patient cohort including patients with recurrence tumour, to better understand the dynamic profiles of EVs and their downstream molecular effect on tumour growth and progression. Future studies should also consider manipulating EVs to selectively target glioma cell growth and progression, such as studying the effect of heparin on GSC-EVs and recipient cells, where heparin can block the transfer of EVs between cells [58]. 

## 4. Methods

### 4.1. Ethical Approval

All glioma stem cell lines used in this study were derived from glioblastoma tissue donated by patients who gave informed written consent. Ethical approval was obtained from the University of Melbourne (UoM) and the Royal Melbourne Hospital (RMH) Ethics Review Committees.

### 4.2. Cell Culture

Three glioma stem cell (GSC) lines were generated from primary human glioblastoma tissues collected at the time of surgery at the Royal Melbourne Hospital and maintained in our ongoing collection of samples within our Brain Tumour Tissue Bank, Department of Surgery. GSCs were cultured as gliomaspheres in serum-free medium (SFM, containing DMEM/F12 + 1% of penicillin/streptomycin + B27 supplement, all from Life Technologies, Carlsbad, CA, USA) + 10 ng/mL epidermal growth factor + 10 ng/mL fibroblast growth factor (both from Miltenyi Biotec, Bergisch Gladbach, Germany) in a T75 cm^2^ ultralow attachment flask (Corning, New York, NY, USA) in a 10% CO_2_ at 37 °C humidified incubator. A total of 10,000 GSCs were seeded, and conditioned media were collected twice weekly for exosome collection.

The human glioma cell lines (LN229 and U118) obtained commercially from ATCC were used as recipient cells for all functional studies. These cells (500 cells/cm^2^) were cultured in a monolayer in DMEM supplemented with 1% of penicillin/streptomycin and 10% of foetal calf-serum (all from Life Technologies) in a 10% CO_2_ at 37 °C humidified incubator. 

### 4.3. Exosome Isolation and Characterisation and Uptake

GSC-derived exosomes/extracellular vesicles (GSC-EVs) isolation and characterisation were performed according to a well-established protocol [59,60]. Briefly, conditioned media (CM) samples were collected from gliomaspheres and then centrifuged at 300× *g* and 2000× *g* for 5 min and 10 min, respectively. The CM samples were further centrifuged at 10,000× *g* for 30 min. Supernatant was then collected and ultracentrifuged at 100,000× *g* for 70 min. EV pellets were concentrated, washed with cold PBS at 100,000× *g* for 70 min and stored at −80 °C for downstream functional studies. 

GSC-EVs uptake was performed according to a previous method with modification [61,62]. Briefly, 1 μM Vybrant DiI lipophilic dye (Life Technologies) was added to GSC-EVs samples and incubated for 10 min at 37 °C. Excess dye was removed by washing with PBS and ultracentrifugation at 100,000× *g* for 70 min at 4 °C. The DiI-labelled EVs were resuspended in 0.2 mL of PBS and overlaid on a 4 mL 30% sucrose cushion (300 g/L of sucrose and 24 g/L of Tris base, pH 7.4), then washed by ultracentrifugation at 100,000× *g* for 70 min at 4 °C. DiI-EVs were harvested at the PBS–sucrose interface and pelleted and stored at −80 °C for the internalisation study. 

For the internalisation experiment, recipient cells (U118 or LN229) were seeded (1 × 10^4^/well overnight) in a CellCarrier 96-well clear black bottom plate (#6005550, PerkinElmer, Waltham, MA, USA) and 10 μL of DiI-EVs was added. The EVs internalisation was monitored and recorded every 10 min for 16 h using an Operetta high-content imaging system (PerkinElmer). A minimum of 3 fields of view for *n* = 3 were recorded and data were processed using Harmony software (v4.1, PerkinElmer) and ImageJ (v1.5.1s, NIH, Bethesda, MD, USA). This experiment was performed at the Biological Optical Microscopy Platform at the University of Melbourne. 

### 4.4. EVs Size and Morphology Quantification

Cryo-electron microscopy (Cryo-EM) was used to record the GSC-EVs size (40–150 nm) and morphology, as previously described [59,62]. Briefly, 3 μL of freshly prepared GSC-EVs was applied to a glow-discharged carbon grid, vitrified and mounted into a Gatan cryo-holder (Gatan, Inc., Pleasanton, CA, USA) and stored in cold liquid nitrogen for analysis. Images of GSC-EVs were acquired using a 300 kV Tecnai G2 F30 system (FEI, Eindhoven, The Netherlands). A minimum of 15 fields of view were recorded per sample. The whole process was repeated at least 3 times. 

Nanoparticle tracking analysis (NTA, NanoSight NS300 instrument, Malvern, UK) was used to record the size and particle concentration of GSC-EVs, using a previously described protocol [28,29]. A sample mixture (100 µL of GSC-EVs: 900 µL of distilled water) was injected into the sample chamber using a 1 mL sterile syringe (BD, Franklin Lakes, NJ, USA), until the first droplet of liquid formed at the tip of the nozzle. Using the NTA software, the optimal field of view (50–100 particle/view) was set and recorded in triplicate for each sample. Data, including graphs of particle size and concentration/mL, were recorded and exported in csv or pdf format. 

### 4.5. Western Blot

Western blotting was used to identify the exosomal protein marker, stem cell marker and PTEN/Akt-associated proteins using standard protocols. All antibodies used are listed in Supplementary Table S1. Briefly, GSC-EVs or recipient cells with or without GSCs-EVs treatment were lysed and quantified using a Pierce BCA Protein Assay Kit (Life Technologies) according to the manufacturer’s instructions. Total protein (10–50 µg) was mixed and incubated with a sample reducing agent and a sample buffer at 95 °C for 5 min and run on 4–12% Bis-Tris gels (all Life Technologies), transferred to 0.45 µm polyvinylidenefluoride membranes (GE Healthcare, Rydelmere, Australia). The membrane was blocked with blocking buffer (5% skim milk in Tris-buffered saline/Tween, TBST) for one hour on a vertical shaker and incubated with primary antibodies overnight at 4 °C. The membranes were washed three times with TBST, for 15 min each. Secondary antibodies (goat anti-rabbit or anti-mouse IgG-HRP, 1:5000, Bio-Rad, Hercules, CA, USA) were added and incubated for one hour at room temperature, and then the wash was repeated. The signal was visualised using an ECL chemiluminescence detection kit (GE Healthcare).

### 4.6. Radiation Treatment 

Recipient cells (LN229 and U118 grown as an adherent monolayer) and GSC neurospheres were irradiated at the optimised dose (2 Gy and 5 Gy for adherent cells; 2 Gy, 4 Gy, 5 Gy and 8 Gy for GSCs neurospheres, with a cumulative exposure time of 0.80–2.50 min and a distance of 50–80 cm, using Theratronics Phoenix cobalt-60 gamma irradiator, Best Theratronics Ltd., Kanata, ON, Canada). Adherent cells (1 × 10^3^ cells/well) were seeded in a 6-well plate for 12 h before irradiation to ensure cell attachment, while GSCs were seeded as single cell suspensions in an ultra-low attachment plate for 18 h prior to irradiation.

### 4.7. Cell Viability Assay

To detect cell proliferation and cell viability, a luminescent readout assay was performed according to the manufacturer’s instructions. In brief, glioma cells (LN229 and U118) with or without GSC-EV treatments were washed twice with PBS, incubated with luciferase reporter assay 1X buffer (Luminescent Cell Viability Assay, Promega, Madison, WI, USA) at room temperature for 15 min and read using a luminometer. Data were normalised to the non-treatment group and presented as a change in ratio. 

### 4.8. Clonogenic Assay

A clonogenic assay was performed according to the previous protocol [63]. Briefly, recipient cells (LN229 and U118) were seeded in the 6-well plate at a concentration of 1 × 10^3^ cells/well in triplicate, allowing for colony formation (7–10 days). Treatments (radiation alone or radiation plus GSC-EVs) were given on the next day of cell seeding and monitored. Cells were washed twice with PBS and incubated with crystal violet mixture (6.0% glutaraldehyde and 0.5% crystal violet) at room temperature for 30 min. Plates were washed twice with tap water, and images of colony formation were taken.

### 4.9. Cell Migration/Invasion

To examine the effect of GSC-EVs on glioma cell migration and invasion, recipient cells (LN229 and U118) were seeded (1 × 10^4^ cells/well) into a 48-well plate and allowed to reach 100% confluency. A scratch was made, and 10 µg of GSC-EVs was added and monitored for 48 h. The cell migration distance was captured at times 0, 24 h and 48 h. The percentage of cell migration was calculated as the ratio of the distance of the migrated cell over time to the distance of the initial scratch × 100. All experiments were performed at least 3 times in triplicate. 

### 4.10. Zymography and Invadopodia Assay

To determine the matrix metalloproteinase activity of GSC-EVs and the treated recipient cells, gelatinase zymographic measurements were made, as described [32]. Briefly, LN229 and U118 were pre-treated with GSC-EVs for 48 h, washed with PBS and cultured in serum-free medium for 24 h prior to collection. Conditioned media and/or GSC-EVs (MU004 EV, MU020 EV and MU039 EV) were diluted with Novex Tris-Glycine SDS sample buffer and separated by electrophoresis at 125 V for 1.5 h on Novex 10% Zymogram (gelatin) gels, followed by incubation with developing buffer for 30 min and incubation overnight at 37 °C, according to the manufacturer’s instructions (Life Technologies). Clear and visible gelatinolytic bands and their molecular weights were identified against the loaded Precision Blue (BioRad, Hercules, CA, USA) protein markers. The gels were then scanned on a flatbed scanner for densitometric analysis using ImageJ (NIH).

To assess the invadopodia and matrix degradation ability, LN229 and U118 recipient cells were prepared by incubating GSC-EVs (MV004 EVs, MV020 EVs, MV039 EVs or untreated) for 48 h, trypsinised and seeded at 7.5 × 10^4^ cells per FITC–gelatin coated coverslip and incubated for 24 h, as described [32,64]. Coverslips were stained with rhodamine phalloidin (dilution 1:75, Cytoskeleton Inc., Denver, CO, USA) and DAPI nuclear staining (5 µg/mL, Sigma-Aldrich, St. Louis, MO, USA), as per the manufacturers’ instructions. Degraded FITC-labelled gelatin was defined as a black area depleted of fluorescent gelatin in images acquired and analysed using a Nikon A1+ confocal microscope system equipped with a Plan Apo VC 60x DIC N2 oil immersion objective and ImageJ (NIH). A total of 10 random images per field of view were captured, and the area of degradation was calculated based on the threshold and region set for the total regions of matrix degradation in a given image field, normalised to the number of DAPI-positive nuclei. 

### 4.11. Exosomes miRNA Profiling and Analysis

The miRNA profiling was performed on GSC-EVs using NanoString (NanoString Technologies, Seattle, WA, USA). Total RNA was extracted from GSC-EVs using an Exiqon miRNA extraction kit according to the manufacturer’s instructions and profiled using NanoString miRNA assay profiling. Data were analysed using nSolver analysis software (v3.0, NanoString Technologies) and the R statistical package. Highly expressed miRNAs were further analysed and compared with the Glioblastoma Bio Discovery Portal (GBM-BioDP, https://gbm-biodp-nci-nih-gov (accessed on 1 December 2021) [65]) for glioma relevance and survival status [65]. It is acknowledged that the data compiled here on the GBM-BioDP were based on the H-miRNA_8 × 15 K platform studied by Verhaak et al. [35,65].

### 4.12. Statistical Analysis

All plots were analysed and generated using GraphPad Prism v7 (GraphPad, San Diego, CA, USA). Student’s *t*-test was used to compare treatment and control groups. Where indicated, one-way or two-way ANOVA was used for multiple-group comparisons, and a post hoc test for individual comparison was then performed when differences were shown using ANOVA. Differences were considered to be statistically significant when the *p*-value was less than 0.05. All miRNA counts were derived from the algorithm formula within the nSolver analysis software (v3.0, NanoString Technologies).

## 5. Conclusions

This study demonstrated that extracellular vesicles are secreted by glioma stem cells (GSC-EVs) to modulate the tumour microenvironment and make the tumour cells become resistant to radiation treatment. GSC-EVs contain a specific set of miRNAs and can promote glioma cell migration, invasion and proliferation, probably via the PTEN/Akt pathway. Currently, we are examining the expression of these miRNAs within the plasma and serum derived from patients, to study their effects on the activation of the PTEN/Akt pathway. Our future aims are to inhibit the GSC-EVs action and to neutralise these GSC-EVs as therapeutic targets. 

## Figures and Tables

**Table 1:** List of the top 25 highly expressed miRNAs in GSC-EVs.

Gene Name	Accession #	NanoString Raw Counts			Sequence	Genome Context	PTEN Position
		MU004 EVs	MU020 EVs	MU039 EVs			
hsa-miR-451a	MIMAT0001631	15,460	23	14	aaaccguuaccauuacugaguu	chr17: 28861369-28861440 [-]	
* hsa-miR-320e	MIMAT0015072	4936	31	30	aaagcuggguugagaagg	chr19: 46709293-46709345 [-]	Position 4736–4742 of PTEN 3′ UTR
* hsa-miR-520f-3p	MIMAT0002830	726	1184	140	aagugcuuccuuuuagaggguu	chr19: 53682159-53682245 [+]	Position 1149–1155 of PTEN 3′ UTR
hsa-miR-873-3p	MIMAT0022717	162	154	123	ggagacugaugaguucccggga	chr9: 28888879-28888955 [-]	
hsa-miR-223-3p	MIMAT0000280	482	24	15	ugucaguuugucaaauacccca	chrX: 66018870-66018979 [+]	
* hsa-miR-363-3p	MIMAT0000707	121	131	92	aauugcacgguauccaucugua	chrX: 134169378-134169452 [-]	Position 2859–2866 of PTEN 3′ UTR
* hsa-miR-144-3p	MIMAT0000436	390	21	16	uacaguauagaugauguacu	chr17: 28861533-28861618 [-]	Position 2917–2923 of PTEN 3′ UTR
hsa-miR-598-3p	MIMAT0003266	117	112	75	uacgucaucguugucaucguca	chr8: 11035206-11035302 [-]	
hsa-miR-6721-5p	MIMAT0025852	124	103	63	ugggcaggggcuuauuguaggag	chr6: 32170030-32170116 [-]	
* hsa-miR-16-5p	MIMAT0000069	214	48	30	uagcagcacguaaauauuggcg	chr3: 160404745-160404825 [+]	Position 4318–4325 of PTEN 3′ UTR
hsa-miR-4443	MIMAT0018961	79	91	56	uuggaggcguggguuuu	chr3: 48196564-48196616 [+]	
^#^ hsa-miR-612	MIMAT0003280	67	65	83	gcugggcagggcuucugagcuccuu	chr11: 65444458-65444557 [+]	
hsa-miR-513b-5p	MIMAT0005788	71	110	25	uucacaaggaggugucauuuau	chrX: 147199044-147199127 [-]	
hsa-miR-1183	MIMAT0005828	71	51	47	cacuguaggugauggugagagugggca	chr7: 21471058-21471146 [+]	
* hsa-miR-495-3p	MIMAT0002817	60	75	41	aaacaaacauggugcacuucuu	chr14: 101033755-101033836 [+]	Position 3221–3227 of PTEN 3′ UTR, Position 3232–3239 of PTEN 3′ UTR
* hsa-miR-23a-3p	MIMAT0000078	145	20	25	aucacauugccagggauuucc	chr19: 13836587-13836659 [-]	Position 1608–1615 of PTEN 3′ UTR, Position 2279–2286 of PTEN 3′ UTR, Position 4753–4760 of PTEN 3′ UTR
hsa-miR-150-5p	MIMAT0000451	184	13	13	cugguacaggccugggggacag	chr19: 49500785-49500868 [-]	
hsa-miR-761	MIMAT0010364	68	63	37	gcagcagggugaaacugacaca	chr1: 51836344-51836402 [-]	
hsa-miR-149-5p	MIMAT0000450	55	55	39	ucuggcuccgugucuucacuccc	chr2: 240456001-240456089 [+]	
hsa-miR-548ah-5p	MIMAT0018972	49	42	39	aaaagugauugcaguguuug	chr4: 76575551-76575626 [+]	
^#^ hsa-miR-142-3p	MIMAT0000434	129	20	16	uguaguguuuccuacuuuaugga	chr17: 58331232-58331318 [-]	
hsa-miR-2682-5p	MIMAT0013517	49	46	49	caggcagugacuguucagacguc	chr1: 98045242-98045351 [-]	
hsa-miR-548al	MIMAT0019024	49	49	46	aacggcaaugacuuuuguacca	chr11: 74399237-74399333 [+]	
^#^ hsa-miR-551a	MIMAT0003214	50	57	40	gcgacccacucuugguuucca	chr1: 3560695-3560790 [-]	
* hsa-miR-155-5p	MIMAT0000646	41	43	53	uuaaugcuaaucgugauaggggu	chr21: 25573980-25574044 [+]	Position 6323–6329 of PTEN 3′ UTR

*: target PTEN. #: Common miRNAs found in GBM Bio Discovery Portal—https://gbm-biodp.nci.nih.gov (accessed on 1 December 2021).

## Data Availability

All figures and data used to support the findings of this study are included within the article or can be made available through the corresponding author upon request.

## Supplementary Materials

The following supporting information can be downloaded at: https://www.mdpi.com/article/10.3390/ijms23052770/s1.
