# Supplementary material for: Extracellular Vesicles Secreted by Glioma Stem Cells Are Involved in Radiation Resistance and Glioma Progression
Source: Int J Mol Sci. 2022 Mar 2;23(5):2770. doi: 10.3390/ijms23052770 (PMC8911495; doi:10.3390/ijms23052770)
Supplement: Supplementary file 1 [file ijms-23-02770-s001.zip › ijms-1602584-SI/Ma et al Supp Figures.pptx]

## Slide 1
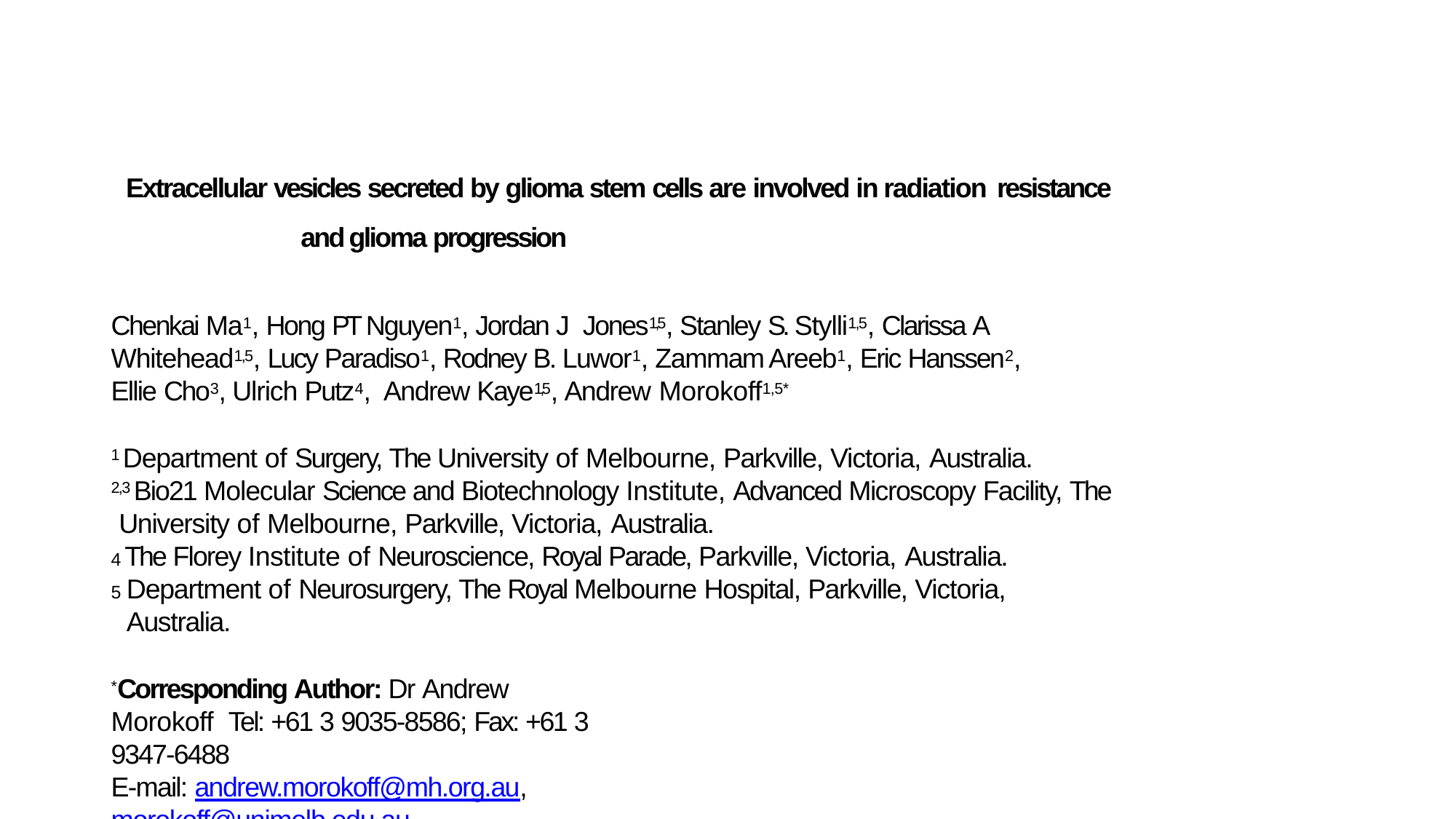

Extracellular vesicles secreted by glioma stem cells are involved in radiation resistance
and glioma progression
Chenkai Ma1, Hong PT Nguyen1, Jordan J Jones1,5, Stanley S. Stylli1,5, Clarissa A Whitehead1,5, Lucy Paradiso1, Rodney B. Luwor1, Zammam Areeb1, Eric Hanssen2, Ellie Cho3, Ulrich Putz4, Andrew Kaye1,5, Andrew Morokoff1,5*
1 Department of Surgery, The University of Melbourne, Parkville, Victoria, Australia.
2,3 Bio21 Molecular Science and Biotechnology Institute, Advanced Microscopy Facility, The University of Melbourne, Parkville, Victoria, Australia.
The Florey Institute of Neuroscience, Royal Parade, Parkville, Victoria, Australia.
Department of Neurosurgery, The Royal Melbourne Hospital, Parkville, Victoria, Australia.
*Corresponding Author: Dr Andrew Morokoff Tel: +61 3 9035-8586; Fax: +61 3 9347-6488
E-mail: andrew.morokoff@mh.org.au, morokoff@unimelb.edu.au

## Slide 2
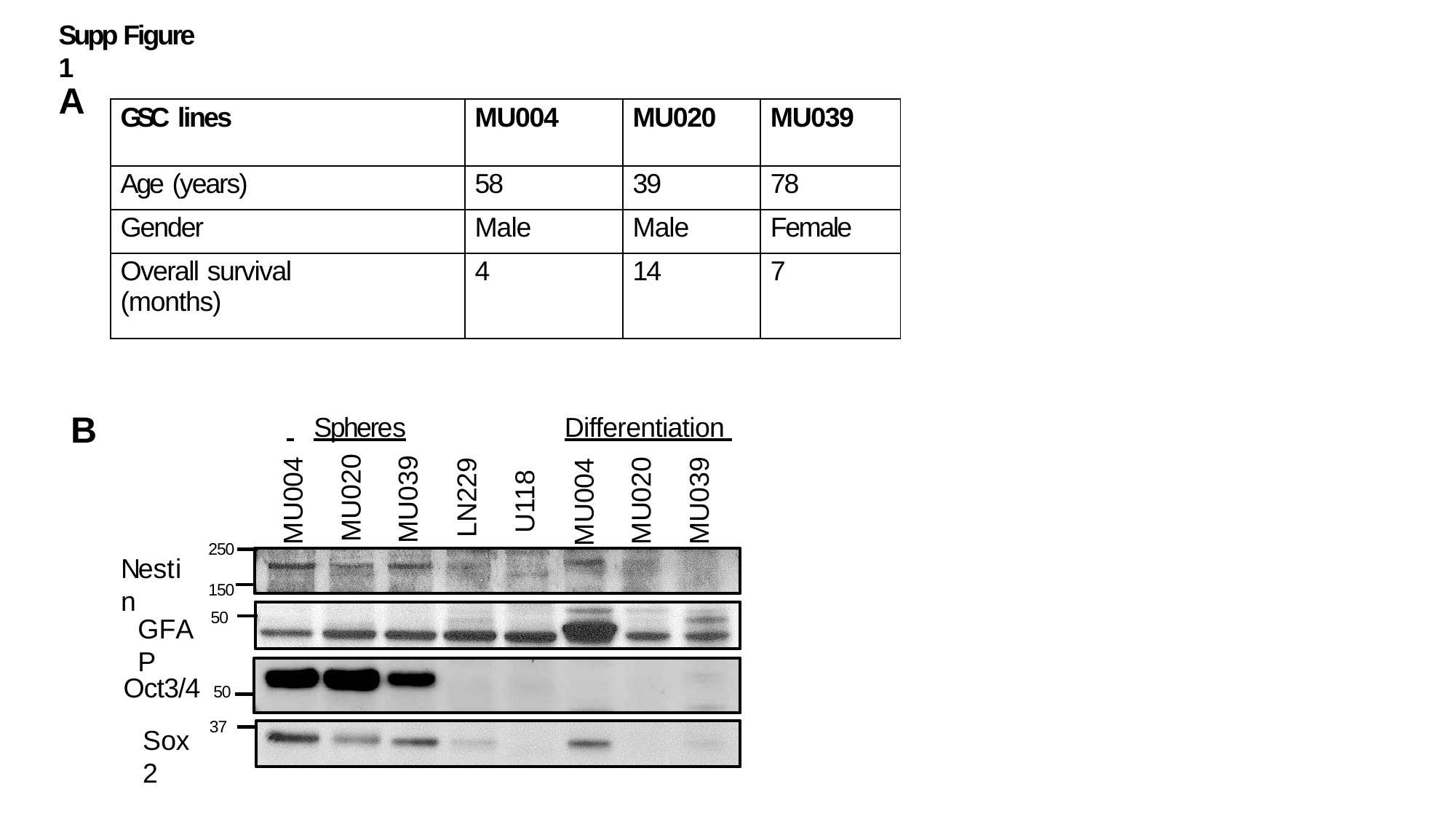

# Supp Figure 1
A
| GSC lines | MU004 | MU020 | MU039 |
| --- | --- | --- | --- |
| Age (years) | 58 | 39 | 78 |
| Gender | Male | Male | Female |
| Overall survival (months) | 4 | 14 | 7 |
B
 	Spheres
Differentiation
MU020
MU039
MU004
MU039
MU004
MU020
U118
LN229
250
Nestin
150
50
GFAP
Oct3/4 50
37
Sox2

## Slide 3
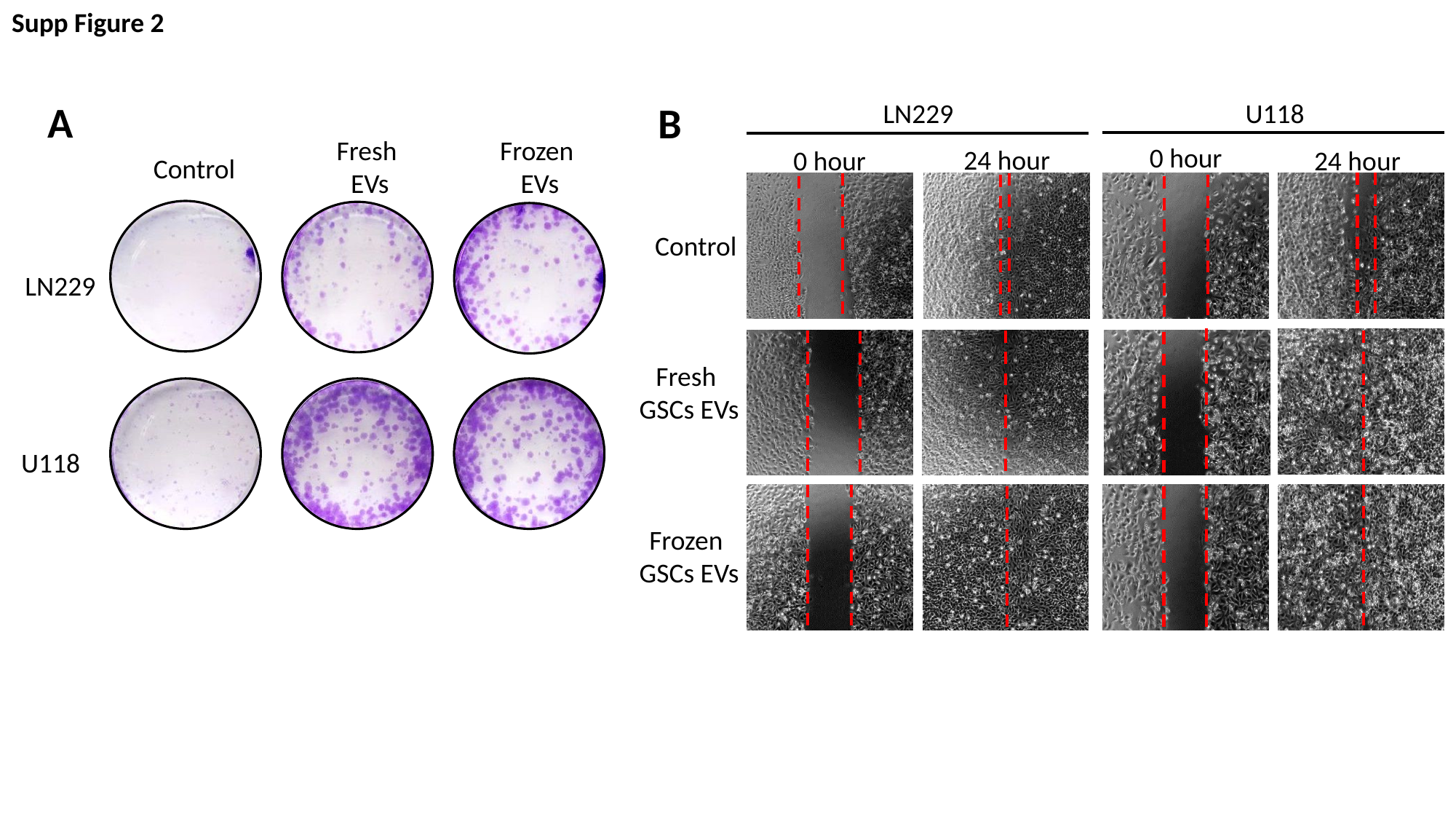

Supp Figure 2
A
Fresh
EVs
Frozen
EVs
Control
LN229
U118
U118
B
LN229
0 hour
24 hour
24 hour
0 hour
Control
Fresh
GSCs EVs
Frozen
GSCs EVs

## Slide 4
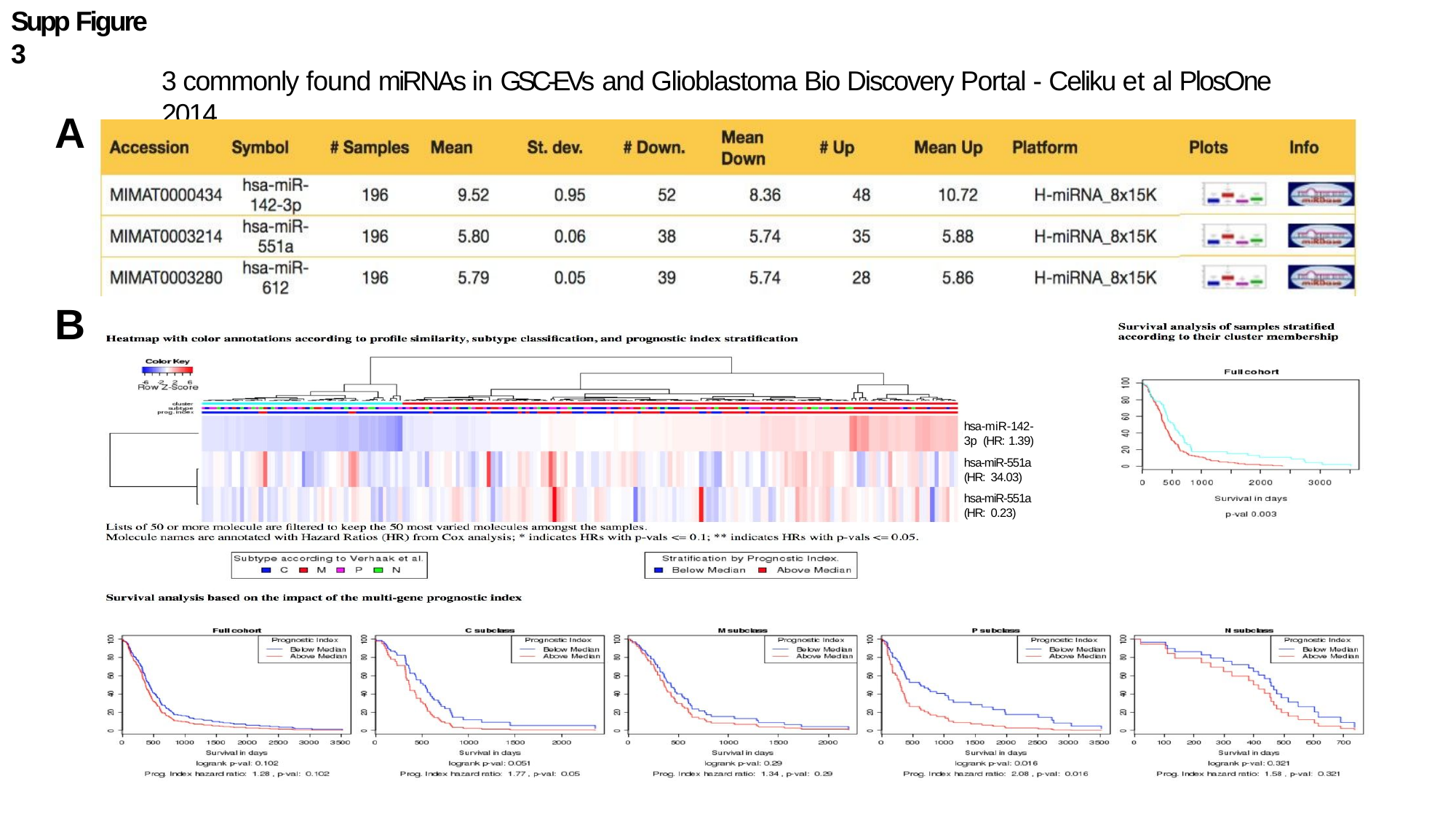

# Supp Figure 3
3 commonly found miRNAs in GSC-EVs and Glioblastoma Bio Discovery Portal - Celiku et al PlosOne 2014
A
B
hsa-miR-142-3p (HR: 1.39)
hsa-miR-551a (HR: 34.03)
hsa-miR-551a (HR: 0.23)
