# Supplementary material for: Extracellular Vesicles Secreted by Glioma Stem Cells Are Involved in Radiation Resistance and Glioma Progression
Source: Int J Mol Sci. 2022 Mar 2;23(5):2770. doi: 10.3390/ijms23052770 (PMC8911495; doi:10.3390/ijms23052770)
Supplement: Supplementary file 1 [file ijms-23-02770-s001.zip › ijms-1602584-SI/Ma et al Supp Legends.pdf]

Extracellular vesicles secreted by glioma stem cells are involved in radiation resistance and glioma progression

Chenkai Ma[1#], Hong PT Nguyen[1#], Jordan J Jones[1,5], Stanley S. Stylli[1,5], Clarissa A Whitehead[1,5], Lucy Paradiso[1], Rodney B. Luwor[1], Zammam Areeb[1], Eric Hanssen[2], Ellie Cho[3], Ulrich Putz[4], Andrew Kaye[1,5], Andrew Morokoff[1,5*]

Supplementary Legends:

Supplementary Figure 1. Characteristic of glioma stem cells in this study. **A)** Clinical information of 3 GSC cell lines: MU004, MU020 and MU039. **B)** Representative protein expression of neuronal markers (Nestin and GFAP) and stem cells markers (Oct3/4 and Sox2) in GSC lines grown as spheres and monolayer (differentiation) non-GSC cells and the recipient cells LN229 and U118.

Supplementary Figure 2: Fresh and frozen GSC-EVs on colony formation. **A)** Representative images of colony formation in LN229 and U118 in the presence of fresh vs. frozen GSC-EVs. **B)** Representative images of cell migration in LN229 and U118 in response to fresh vs. frozen GSC-EVs.

Supplementary Figure 3: Commonly found miRNAs between GSC-EVs and public database. **A)** 3 GSC-EVs packaged miRNAs (hsa-miR-142-3p, hsa-miR551a and hsa-miR612) were found in common with Celiku et al within the Glioblastoma Bio-Discovery Portal indicating their involvement in glioblastoma cellular phenotype and progression. **B)** Heatmap classification of these miRNAs into glioma subtypes and their respective survival analysis.

Supplementary Video 1: Representative live cell recording of GSC-EVs (green labelled) uptake by recipient cells (red labelled) and participated in the cell division and residing in the daughter cells.

Supplementary Table 1: List of antibodies used in this study.

Supplementary Table 2: Common miRNAs found between our current study and other studies that are involved in glioma progression.
